# The human vestibular cortex: functional anatomy of OP2, its connectivity and the effect of vestibular disease

**DOI:** 10.1093/cercor/bhac085

**Published:** 2022-03-02

**Authors:** Richard T Ibitoye, Emma-Jane Mallas, Niall J Bourke, Diego Kaski, Adolfo M Bronstein, David J Sharp

**Affiliations:** Computational, Cognitive and Clinical Neuroimaging Laboratory, Department of Brain Sciences, Imperial College London, London W12 0NN, United Kingdom; Neuro-otology Unit, Department of Brain Sciences, Imperial College London, London W6 8RP, United Kingdom; Computational, Cognitive and Clinical Neuroimaging Laboratory, Department of Brain Sciences, Imperial College London, London W12 0NN, United Kingdom; UK Dementia Research Institute, Care Research & Technology Centre, Imperial College London, London W12 0BZ, United Kingdom; Computational, Cognitive and Clinical Neuroimaging Laboratory, Department of Brain Sciences, Imperial College London, London W12 0NN, United Kingdom; Department of Clinical and Motor Neurosciences, Centre for Vestibular and Behavioural Neurosciences, University College London, London WC1N 3BG, United Kingdom; Neuro-otology Unit, Department of Brain Sciences, Imperial College London, London W6 8RP, United Kingdom; Computational, Cognitive and Clinical Neuroimaging Laboratory, Department of Brain Sciences, Imperial College London, London W12 0NN, United Kingdom; UK Dementia Research Institute, Care Research & Technology Centre, Imperial College London, London W12 0BZ, United Kingdom; Centre for Injury Studies, Imperial College London, London SW7 2AZ, United Kingdom

**Keywords:** perception, visual, vestibular neuritis, vestibular cortex

## Abstract

Area OP2 in the posterior peri-sylvian cortex has been proposed to be the core human vestibular cortex. We investigated the functional anatomy of OP2 and adjacent areas (OP2^+^) using spatially constrained independent component analysis (ICA) of functional magnetic resonance imaging (fMRI) data from the Human Connectome Project. Ten ICA-derived subregions were identified. OP2^+^ responses to vestibular and visual motion were analyzed in 17 controls and 17 right-sided vestibular neuritis patients who had previously undergone caloric and optokinetic stimulation during fMRI. In controls, a posterior part of right OP2^+^ showed: (i) direction-selective responses to visual motion and (ii) activation during caloric stimulation that correlated positively with perceived self-motion, and negatively with visual dependence and peak slow-phase nystagmus velocity. Patients showed abnormal OP2^+^ activity, with an absence of visual or caloric activation of the healthy ear and no correlations with vertigo or visual dependence—despite normal slow-phase nystagmus responses to caloric stimulation. Activity in a lateral part of right OP2^+^ correlated with chronic visually induced dizziness in patients. In summary, distinct functional subregions of right OP2^+^ show strong connectivity to other vestibular areas and a profile of caloric and visual responses, suggesting a central role for vestibular function in health and disease.

## Introduction

The cortical vestibular system is fundamental to self-motion perception and balance (Cullen 2019). Area OP2 in the posterior peri-sylvian region is a core vestibular area in humans (Eickhoff, Schleicher, et al. 2006b; Eickhoff, Weiss, et al. 2006c; Lopez et al. 2012; zu Eulenburg et al. 2012), but the functional organization of OP2 and immediately adjacent areas (OP2^+^) remains unclear.

**Fig. 1:**
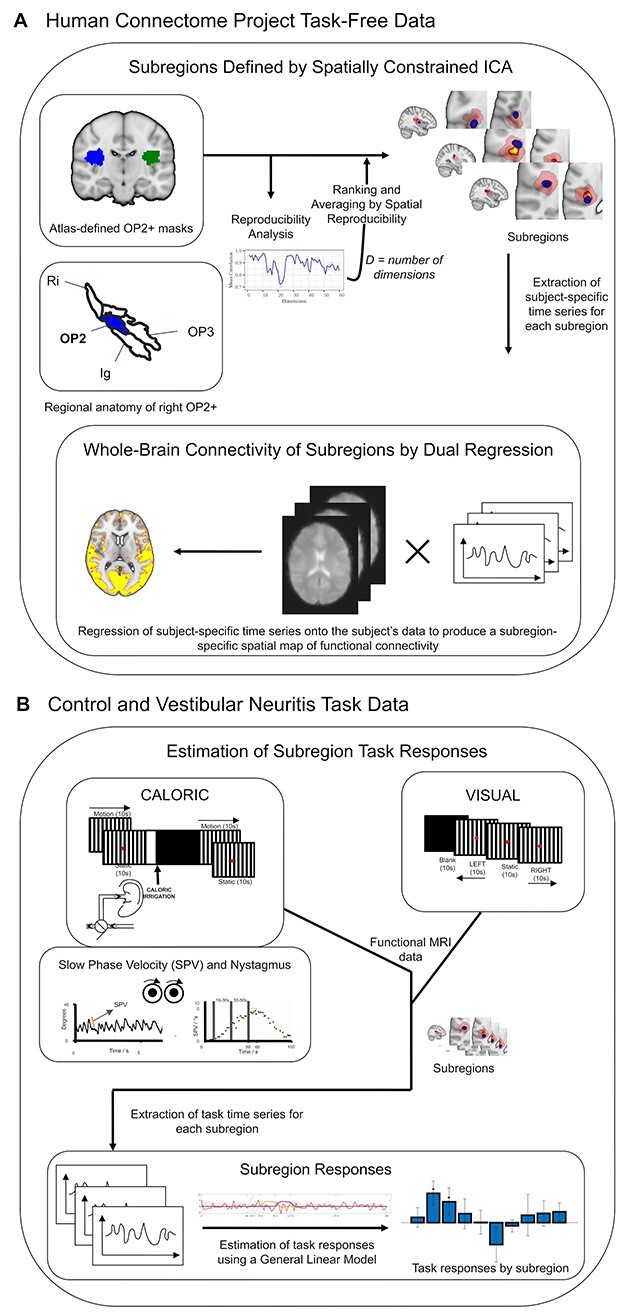
Overview of methods. (A) Spatially constrained ICA of area OP2^+^ within Human Connectome Project task-free data produced spatial maps of subregions in each hemisphere. Between-subject reproducibility was maximized and reproducibility was further enhanced by ranking and averaging by spatial reproducibility. The whole-brain connectivity of each subregion was then estimated by dual regression. The regional anatomy of right OP2^+^ (in blue) is shown on an inflated brain relative to areas OP1, OP3, the retroinsular cortex (Ri), and the insular granular cortex (Ig); the images are adapted with permission from Indovina et al. (Indovina et al. 2020) (B) CALORIC and VISUAL tasks were performed. The trace on the left is an example of right-beating nystagmus during cool caloric irrigation of the left ear; the orange line depicts SPV, which is defined as the gradient of the slow phase of nystagmus; the adjacent trace is a typical profile of the evolution of SPV over time following caloric irrigation at time zero; the time periods (10–30 and 30–50 s) that define the caloric contrast are illustrated.

Area OP2 is located in the posterior parietal operculum and extends into the retroinsular (posterior insular) cortex (Fig. 1A) (Eickhoff, Weiss, et al. 2006c). Meta-analyses have shown consistent activation in OP2 following vestibular stimulation (Lopez et al. 2012; zu Eulenburg et al. 2012). OP2 is homologous to the nonhuman primate parieto-insular vestibular cortex (PIVC)—a “core” cortical vestibular region (Eickhoff, Weiss, et al. 2006c) receiving vestibular, visual, and somatosensory inputs (Guldin and Grüsser 1998). Neural activity in nonhuman primate PIVC encodes head motion (Grüsser et al. 1990a, 1990b; Chen et al. 2010) and is necessary for accurate perception of self-motion (Chen et al. 2016).

Areas neighboring OP2 respond to visual as well as vestibular stimuli (Brandt et al. 1998; Sunaert et al. 1999; Frank et al. 2014; Frank, Wirth, et al. 2016b). The retroinsular cortex is proposed to integrate visual and vestibular motion information, whereas more anterior areas in OP2 process vestibular information (Frank and Greenlee 2018). The processing of visual and vestibular information is affected by unilateral vestibular failure, e.g. vestibular neuritis—a major cause of acute vertigo. Despite central adaptation, many patients are left with long-term vertigo (Imate and Sekitani 1993; Cousins et al. 2014) and the failure to recover fully is accompanied by over-reliance on visual information for perceptual and balance judgments (visual dependence) (Kanayama et al. 1995; Bronstein and Dieterich 2019). The neural basis of this change is unknown but based on data showing an association between gray matter volume in OP2 and symptom load (Helmchen et al. 2009), and altered functional connectivity in OP2 neighboring regions following vestibular neuritis (Helmchen et al. 2014), we propose neural activity within OP2 is relevant to multisensory processing.

An understanding of the vestibular function of OP2 and immediately adjacent areas (herein together referred to as OP2^+^) could be informed by studying its functional connectivity. Structurally connected regions often show correlated brain activity, which reflects shared functions (Smith et al. 2009). Heteromodal brain areas show functional connectivity, which echoes that of whole-brain intrinsic connectivity networks, consistent with a complex subregional structure (Leech et al. 2012; Braga et al. 2013). Subregions with different functions can be identified by their distinct patterns of functional connectivity using spatially constrained independent component analysis (ICA) (Leech et al. 2011, 2012; Moher Alsady et al. 2016). This has been an informative approach for understanding the functions of a range of brain regions (Leech et al. 2012; Moher Alsady et al. 2016; De Simoni et al. 2018), but the method has never before been applied to the vestibular cortex.

Thus, the main aim of this study is to define the functional anatomy of OP2^+^ in a data-driven way using spatially constrained ICA applied to Human Connectome Project (HCP) data (Glasser et al. 2013). In addition, using data collected during a previous study on visual–vestibular activation (Roberts et al. 2018) we investigate OP2^+^ responses in controls and patients with chronic vestibular neuritis. We then correlate these functional magnetic resonance imaging (fMRI) responses with behavioral measures. We test whether OP2^+^ activation during caloric irrigation and visual motion stimulation is homogeneous or restricted to specific functional subregions. We determine the relationship between vestibular activation in subregions of OP2^+^ (defined by distinct functional connectivity) and vertigo, visual dependence, and clinical outcome following vestibular neuritis.

## Materials and methods

In this work, we refer to “subregions” of OP2^+^. Those defined by applying ICA to HCP data are referred to as “ICA-derived.” Subregions that activate in response to caloric vestibular stimulation are referred to as “caloric-responsive.”

## Parcellation of OP2^+^ by functional connectivity

### Participants, MRI acquisition, and preprocessing

The HCP is an openly accessible dataset of high-quality neuroimaging data (Glasser et al. 2013). Magnetic resonance imaging (MRI) data from 100 healthy unrelated participants (54 females, aged 22–36 years) within the HCP were used.

HCP MRI data had been collected on a 3 T Siemens Connectome Skyra scanner and included a T1-weighted magnetization prepared rapid gradient echo image and a resting-state fMRI sequence for each participant (Glasser et al. 2013). HCP MRI data had been optimally preprocessed by the HCP consortium with FMRIB Software Library (FSL) and Freesurfer (version 52) tools. Task-free functional (echo-planar) MRI data were registered to Montreal Neurological Institute (MNI) standard space in a 2-step process; a linear transform of the participant’s echo-planar image to their structural (T1-weighted) image was combined with a nonlinear transform of the participant’s structural image to the standard (MNI) structural image (Jenkinson et al. 2002). A high-pass filter (2000 s) was then applied. Artifacts were removed using FMRIB’s ICA-based X-noisifier (FIX) (Salimi-Khorshidi et al. 2014). FIX classifies components and regresses out noise and motion-related signals.

## MRI analysis

### Echoes of whole-brain intrinsic connectivity networks in OP2^+^

We summarized the resemblance between functional connectivity within OP2^+^ and whole-brain intrinsic connectivity networks in the HCP dataset using an established method (Leech et al. 2012; Braga et al. 2013). Functional connectivity in OP2^+^ (see definition in Spatially constrained ICA) was fractionated into 10 components using ICA (Braga et al. 2013). The whole-brain connectivity map of each independent component of OP2^+^ was then spatially correlated against 20 whole-brain intrinsic connectivity networks derived from ICA applied to the HCP data (Leech et al. 2012). The number of OP2^+^ components with clear spatial correlation (*r* > 0.3) with one or more intrinsic connectivity networks was a summary measure. Heteromodality was suggested by the presence of more than one component that echoed the connectivity of whole-brain intrinsic connectivity networks.

### Spatially constrained ICA

Spatially constrained ICA was applied to OP2^+^ in the HCP task-free fMRI dataset, using the masked ICA (mICA) toolbox (Moher Alsady et al. 2016). Right and left OP2^+^ masks were created using the Jülich probabilistic atlas within the FSL (Eickhoff, Heim, et al. 2006a). The masks were not thresholded to ensure immediately adjacent parietal opercular and retroinsular areas were included. Masked participant data was spatially smoothed using a 5-mm kernel prior to ICA.

### Split-half sampling

To select an optimum dimensionality for the ICA decomposition in a data-driven way, we undertook split-half sampling as implemented in the mICA (Moher Alsady et al. 2016). This method maximizes between-subject reproducibility. The analysis of between-subject reproducibility involved partitioning the HCP participants into two-halves by random sampling, repeated 50 times. Group ICA was then undertaken for each of the two-halves over 1–60 dimensions, for each sampling repetition. Independent components with similar functional connectivity were matched by the Pearson correlation coefficient of their time series between the 2 split-halves. The mean correlation of matched time series pairs across repetitions was the measure of reproducibility. As recommended, we selected the dimensionality with the highest global reproducibility (Moher Alsady et al. 2016). The ideal number of dimensions for ICA was first established in right OP2^+^. Left OP2^+^ was then decomposed into the same number of subregions. The motivation for starting with right OP2^+^ is the meta-analysis evidence that shows right OP2 is core to the cortical vestibular response (zu Eulenburg et al. 2012).

### Ranking and averaging ICA by reproducibility

ICA results are unstable across repeat decompositions (realizations), limiting reproducibility (Himberg et al. 2004). We therefore applied an established method where the results of multiple realizations were ranked and averaged by their spatial reproducibility (Ranking and Averaging Independent Component Analysis by Reproducibility, RAICAR; Yang et al. 2008 source code available at https://github.com/yangzhi-psy/RAICAR). ICA was repeated 30 times for the dimensionality that maximized between-subject reproducibility. Components from each realization were then iteratively matched with the most similar components from other realizations, by maximizing spatial correlation. The results of this process were distinct, aligned component sets. Each set contained members from different realizations. For each aligned component set, a cross-realization spatial cross-correlation matrix was then produced. A default cut-off spatial correlation of 0.5 was used in this study to identify reproducible components (Yang et al. 2008). Finally, for each aligned component set, a selective average of the spatial maps and time series of the members across realizations was produced. Only realizations that had at least one spatial correlation coefficient higher than the cut-off threshold were included in this selective average.

### Dual regression

We investigated the whole-brain connectivity of ICA-derived OP2^+^ subregions within the HCP participant data. To do this, we applied dual regression within FSL (Fig. 1A) (Beckmann et al. 2009; Nickerson et al. 2017). This technique generates participant-specific spatial maps of functional connectivity using an initial spatial map (here OP2^+^ subregions) and participant fMRI data. First, for each participant, the OP2^+^ subregion spatial maps were regressed (as spatial regressors in a multiple regression) into the participant’s fMRI data. This produced a participant-specific time series for each subregion, with the influence of other subregions partialled out. Next, for each participant, the resultant time series is then regressed onto the same fMRI data to produce participant-specific spatial maps of functional connectivity for each subregion (Nickerson et al. 2017). Finally, following dual regression, group-level whole-brain connectivity maps for each subregion were derived by applying nonparametric permutation testing to the spatial map outputs of dual regression. Permutation testing was done using randomize (within FSL, *n* = 10,000, *P* < 0.05) (Winkler et al. 2014). We tested for the inclusion of known regions of interest from zu Eulenburg et al.’s meta-analysis of vestibular stimulation studies, as evidence of vestibular connectivity (zu Eulenburg et al. 2012).

### Spatial similarity of whole-brain connectivity by hierarchical clustering

We investigated the spatial similarity between the whole-brain connectivity maps of the ICA-derived subregions of OP2^+^ using hierarchical clustering analysis (implemented in MATLAB). Spatial correlation between the whole-brain connectivity maps for each pair of subregions was determined. This correlation matrix was transformed to a pairwise Euclidean distance matrix (*pdist* function), then the distances were used to generate an agglomerative hierarchical cluster tree (*linkage* function using the default “single” method). This output was used to infer clustering by spatial similarity.

## Task-based functional activity in controls and in patients with vestibular neuritis

### Participants

Seventeen right-handed patients with chronic (> 6 months since symptom onset) right-sided vestibular neuritis participated in the original study (mean age 58.8 ± 17.3 [standard deviation, SD] years, 9 females) (Roberts et al. 2018). They presented acutely with vertigo. Examination revealed left-beating horizontal jerk nystagmus and catch-up saccades following head impulse tests to the right (Halmagyi and Curthoys 1988). Caloric testing at diagnosis confirmed paresis of the right horizontal canal (mean 53.1%, SD 34.1%). Patients had no other vestibular symptoms or pre-existing vestibular or neurological disorders. Audiometry was normal for age. Symptoms associated with disability were quantified using questionnaire scores. The burden of dizziness handicap, the frequency of dizziness symptoms and the extent of visually induced dizziness were captured by the Dizziness Handicap Inventory (DHI), Vertigo Symptom Scale (VSS), and Situational Vertigo Questionnaires (SVQ), respectively (Jacob et al. 1989; Jacobson and Newman 1990; Yardley et al. 1992). In the original study (Roberts et al. 2018) 17 right-handed healthy age-matched controls were recruited (mean age 58 ± 14 [SD] years, 10 females). They had no history of vestibular or neurological disorders. Caloric vestibular test results were normal. Written informed consent was obtained from all participants. All procedures were performed in accordance with the ethical standards of the Bromley and the Fulham Local Research Ethics Committee.

### Psychophysical measures

All participants had a screening caloric test. The magnitude of vertigo (a sensation of self-motion when no self-motion is occurring) experienced during this screening served as a reference for subsequent vertigo ratings following caloric irrigation in each fMRI session. The magnitude of the experience was reported using a Likert scale from 0 to 10 (higher is more vertigo), with “5” representing the magnitude experienced during screening. A higher score represented more vertigo. These ratings reflected the intensity of perceived self-motion immediately after each caloric task fMRI session.

The subjective visual vertical—a judgment on “which way is up”—depends on otolithic vestibular, visual, and somatosensory signals. Visual dependence—the extent to which the subjective visual vertical is biased by background visual motion—was measured outside the scanner (Fig. 7C (ii)) (Cousins et al. 2014; Roberts et al. 2017). Here, the perceived vertical, the subjective visual vertical, was first measured in a static visual surround. This was then repeated in the presence of background visual motion (Fig. 7C (ii)). Participants looked at a computer screen through a 30-cm deep viewing cone while standing. A 6-cm white rod was presented on a black background. Around a central 6-cm circle was a field of 220 randomly distributed off-white dots each subtending 1.5 degrees of visual field. The rod was rotated to a random angle then participants were asked to re-align it to their subjective visual vertical using keyboard controls. The procedure was then repeated during clockwise and counterclockwise rotation of the disc. Each condition was repeated 6 times, then results were averaged. Visual dependence was defined as the absolute bias of verticality estimates in the motion conditions minus the subjective visual vertical with a static background.

### MRI acquisition and preprocessing

Anatomical and fMRI data from patients with vestibular neuritis and controls were acquired on a Siemens Verio 3-T scanner using standard procedures (Roberts et al. 2017). Echo-planar functional T2^*^-weighted imaging data were acquired in 44 axial planes using a gradient echo sequence (interleaved order, time repetition [TR] 2500 ms, time echo [TE] 30 ms, flip angle 80 degrees, voxel dimension 3 × 3 × 3 mm, acquisition matrix 64 × 64). Foam pads were used to minimize head movement. T1-weighted images were also acquired (TR 2300 ms, TE 3 ms, TI 900 ms, flip angle 9 degrees, bandwidth 238 Hz/pixel, voxel dimensions 1 × 1 × 1 mm, matrix size 256 × 192, FOV 240 × 256 mm, number of excitations = 1).

MRI data from patients with vestibular neuritis and controls were preprocessed using FSL tools. Structural images were brain-extracted using the Brain Extraction Tool (Smith 2002). Echo-planar images were registered to MNI standard space in a 2-step process; a linear transform of the participant’s echo-planar image to their structural (T1-weighted) image was combined with a nonlinear transform of the participant’s structural image to the standard (MNI) structural image (Jenkinson et al. 2002). Images were spatially smoothed (6-mm full-width at half-maximum Gaussian kernel) then high-pass filtered (100 s). Motion was addressed by motion-correction and denoising using ICA. Estimates of motion within echo-planar images were determined to 6 degrees of freedom using MCFLIRT within FSL (Jenkinson et al. 2002). These estimates were then applied as nuisance regressors to derive motion-corrected images. Preprocessed MRI data were also decomposed using ICA, and motion components were removed using an ICA-based tool for the Automatic Removal of Motion Artifacts (Pruim et al. 2015). Data were pre-whitened prior to analysis within general linear models (Woolrich et al. 2001).

### Caloric and visual tasks

Patients with vestibular neuritis and controls underwent: (i) a caloric protocol (4 conditions of caloric irrigation flanked by visual stimulus blocks) and (ii) a visual localizer task (Roberts et al. 2018). The caloric protocol consisted of cool or warm caloric irrigation in the left ear flanked by 60-s visual motion stimulus blocks with leftward or rightward motion (Fig. 1B), giving a total of 4 conditions. The visual localizer task was performed during a separate fMRI session (Fig. 1B) and consisted of a 40-s block of stimuli including left or right moving gratings, repeated 6 times, totalling 240 s. A description of the tasks is presented in Figure 1B.

### Caloric protocol

Participants were instructed to keep their eyes open throughout. The direction of visual motion stimuli within each block was either left or right. The first visual motion stimulus block comprised 10 s of a horizontally moving vertical grating, followed by 10 s of no motion (“static”), repeated 3 times, during which participants were instructed to look at a central fixation dot subtending 0.5 degrees. The grating was composed of black and white vertical bars. Each vertical stripe subtended an angle of 1.9 degrees, with the whole screen subtending a total angle of 15 degrees. During periods of motion the visual grating moved leftward or rightward at 8 degrees per second. After the visual motion stimulus block, there were 5 s of a written instruction: “Get Ready,” then a 2.5-s written instruction to “turn on the tap,” following which participants initiated caloric irrigation by turning a tap by hand (Fig. 1B) (Roberts et al. 2017, 2018). The irrigation lasted 50 s. During irrigation, the screen was black. A total of 250 mL of cool (30 °C) or warm (44 °C) water flowed into the left external auditory canal. Irrigation was followed by a second visual motion stimulus block of the same composition as the first. Eye movements were recorded throughout by an infrared MRI-compatible eye tracker (Ober consulting, Poland). Vestibular nystagmus slow-phase velocity (SPV) was measured during the final 20 s of each caloric irrigation (when nystagmus is maximal; Guzman-Lopez et al. 2011) to derive a peak slow-phase nystagmus velocity.

The direction of slow-phase eye movements induced by caloric irrigation depends on caloric temperature such that a warm stimulus in the left ear causes the eyes to drift rightwards. The direction of the final visual block’s motion stimuli combined with warm or cool caloric stimuli to produce 4 conditions that were either congruent or incongruent regarding the consistency of the directions of slow-phase eye movements and visual motion (Congruent: cool irrigation + left visual motion [CL], and warm irrigation + right visual motion [WR]; Incongruent: cool irrigation + right visual motion [CR], and warm irrigation + left visual motion [WL]) (Roberts et al. 2017, 2018).

### Visual localizer task

The visual localizer task was performed during a separate fMRI session (Fig. 1B). A 40-s block of stimuli was repeated 6 times, totalling 240 s. Each stimulus block consisted of 10 s of a black screen followed by 10 s of a leftward moving black and white vertical grating, 10 s a static grating, then finally 10 s of a rightward moving grating (Roberts et al. 2017, 2018). The visual grating had the same characteristics as that used in the caloric protocol visual motion stimulus blocks.

## MRI analysis

### Caloric and visual task responses

We investigated the response of OP2^+^ subregions during caloric stimulation using an established approach (Leech et al. 2012; Braga et al. 2013). Here, the time series unique to each subregion was determined. To do this, each ICA-derived subregion was spatially regressed onto caloric task session data with the remaining subregions as nuisance covariates—the first stage of dual regression within FSL (Beckmann et al. 2009; Nickerson et al. 2017). The resulting subregion-specific time series for each session then served as inputs to general linear models to estimate responses using contrasts (Leech et al. 2012).

Mirroring the original methodology applied to this data, vectors representing the onsets of visual motion, visual static, and caloric irrigation were convolved with a double-gamma hemodynamic response function and its temporal derivative (Roberts et al. 2017). Peak vestibular activation was modeled using a contrast of vectors representing the last 20 s of caloric irrigation and the previous 20 s (Fig. 1B); importantly, there was no visual stimulation during this period, so the contrast represents the caloric vestibular response. This approach controlled for the effects of auditory and somatosensory activation while avoiding contamination from movement in the first seconds after caloric onset (note: participants self-triggered the caloric irrigation by turning a tap). In the current study, wherein the aim was to estimate the cortical response to vestibular stimulation, the contrast of interest was the caloric response (which precedes and is independent of the second visual stimulus block, Fig. 1B). Consequently, as the post-caloric visual stimulus was not of interest, information from the 4 conditions were combined to produce a summary caloric response for each participant. For each participant, results across their 4 sessions were transferred to higher level models by a summary statistic approach (Beckmann et al. 2003), modeling subregion responses at the participant level, and at the group-level adjusted for linear effects of age and sex.

To determine the response of OP2^+^ during caloric irrigation, we undertook a whole-brain analysis of the control caloric task dataset within FSL, here using a higher-level mixed effects (FLAME 1 + 2) model to estimate the group response (Woolrich et al. 2001). Clusters were inferred from the resultant z-statistic images, applying a threshold of *z* > 3.1 (*P* < 0.001) and a corrected cluster significance threshold of *P* < 0.05. This activation was compared with a meta-analysis-derived mask of voxels, wherein at least one study showed significant activation to vestibular stimulation (Fig. 5) (Lopez et al. 2012). Meta-analysis mask data were provided courtesy of Dr Christophe Lopez, Aix-Marseille Université (Marseille, France).

A role in integrating visual and vestibular information implies ICA-derived OP2^+^ subregions may encode the direction of visual motion stimuli. Subregion activity during visual motion stimuli might therefore vary with motion direction. To test this, we analyzed the visual task dataset, defining contrasts of visual motion (rightwards minus static, leftwards minus static) and motion direction (rightward minus leftward). Here, as in the analysis of caloric task data, OP2^+^ subregions derived from HCP data were spatially regressed onto participant data to produce subregion-specific time series. The time series were then analyzed within a general linear model to estimate responses linked to visual motion and motion direction.

Corrections for multiple comparisons were undertaken by controlling the False Discovery Rate (FDR) across the subregions within each hemisphere, with a significance threshold of *P* < 0.05 (Genovese et al. 2002).

## Correlation between caloric responses and vestibular functions

We investigated whether self-motion perception, peak nystagmus SPV, and/or visual dependence correlated with ICA-derived OP2^+^ subregion responses during caloric irrigation. As vertigo ratings were obtained after each session, but sessions differed by stimulus condition (cool or warm caloric irrigation combined with left or right visual motion during the final visual block), the relationship between the summary caloric responses and vertigo ratings was estimated using linear mixed-effects regression (using *fitlme* within MATLAB). To visualize results, “adjusted vertigo” was determined. Adjusted vertigo captured the relationship between post-caloric vertigo ratings and the caloric response while averaging out the contributions of other predictors in the linear model (MATLAB) (MATLAB 2021).

To understand the correspondence between self-motion perception (post-caloric vertigo) and nystagmus responses, we investigated the relationship between these variables. The peak nystagmus SPV was measured during each caloric task session from eye movement traces. The relationship between peak nystagmus “SPV” and “vertigo” was investigated using linear mixed-effects regression. “Vertigo” was the dependent variable, with “SPV” and “condition” as fixed effects, and “participant” as a random effect (in a random intercept model).

In patients with vestibular neuritis, we tested for correlation between questionnaire scores of the burden of chronic symptoms and the summary caloric response of OP2^+^ subregions, to identify subregions that may be relevant to clinical recovery following vestibular neuritis.

### Data availability

Raw data which support these results were collected at Imperial College London. These data and derived data supporting the findings in this study are available from the corresponding authors upon reasonable request.

## Results

We were interested in looking at area OP2 and immediately adjacent regions (OP2^+^) as a potential vestibular hub. We therefore first examined its connectivity before looking into its responses to vestibular stimulation.

### OP2^+^ subregions show functional connectivity with other vestibular regions

**Fig. 2:**
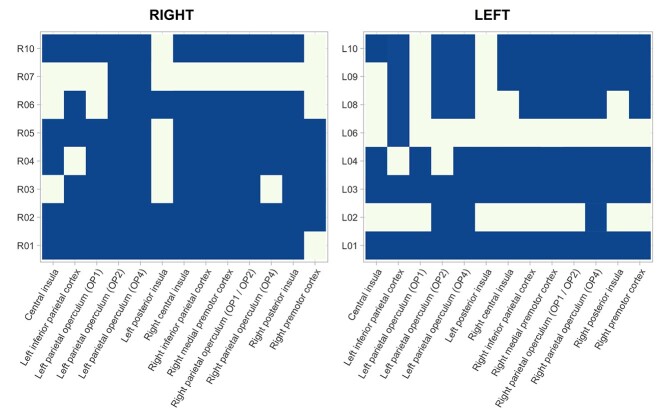
OP2^+^ Subregions are connected to known vestibular areas. Blue-shaded cells indicate significant functional connectivity between OP2^+^ subregions and areas that consistently activated following vestibular stimulation as reported in zu Eulenburg et al’.s (zu Eulenburg et al. 2012) meta-analysis. Family-wise error rate corrected 1 minus *P* values are illustrated, as determined by nonparametric permutation testing of participant and subregion-specific whole-brain connectivity maps using *randomize*, within FSL (Winkler et al. 2014). A value of > 0.95 is considered significant. Cells with nonsignificant connectivity are in the background color.

**Fig. 3:**
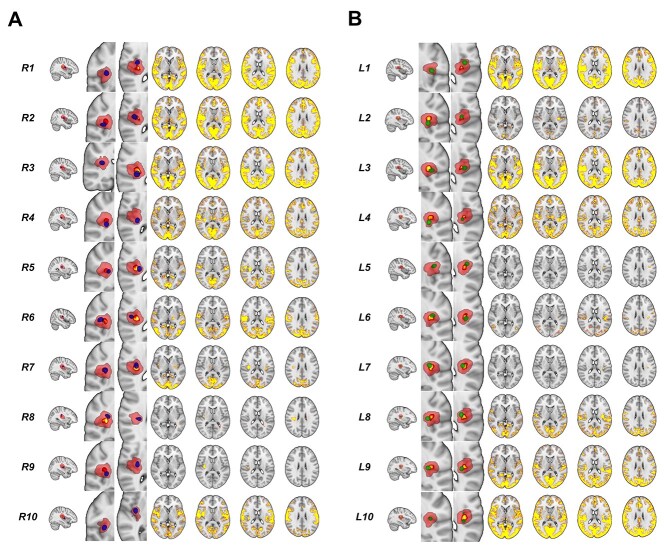
Subregions within OP2^+^ identified from ICA. (A) Right OP2^+^ subregion images (blue) overlaid on right OP2^+^ mask (red, from Jülich atlas). The core/central area of the OP2^+^ mask (*P* > 0.5 in Jülich atlas) is shown in yellow. Brain networks with significant functional connectivity to each OP2^+^ subregion are shown adjacent (significant voxels red-yellow, *P* < 0.05). (B) Left OP2^+^ subregions are shown in green with adjacent functional connectivity maps. R = right. L = left.

Connectivity within right OP2^+^ echoed multiple whole-brain intrinsic connectivity networks, consistent with a complex subregional structure (4 components showed spatial correlation *r* > 0.3). We identified distinct subregions within OP2^+^ on the basis of their functional connectivity using spatially constrained ICA applied to task-free fMRI data from the HCP. Constraining ICA to produce 10 components maximized their reproducibility (*r* = 0.976, SD 0.112). Ten subregions with distinct functional connectivity were identified on the right (Fig. 3A). A range of connectivity patterns were observed (summarized in Fig. 2). Most OP2^+^ subregions were connected with a number of areas previously shown to activate following vestibular stimulation in a meta-analysis (zu Eulenburg et al. 2012), including the insula, parietal operculum, inferior parietal cortex and premotor cortex (Figs 2 and 3A). Overall, 7 of 10 subregions in right OP2^+^ were connected to more than half of regions identified in the meta-analysis (R1, R2, R3, R4, R5, R6 and R10, Fig. 2) (zu Eulenburg et al. 2012). R7 largely connected to the occipital cortex. Subregions R8 and R9 had no meaningful gray matter connectivity.

Ten ICA-derived subdivisions in left OP2^+^ were identified in a similar way. Most subregions in left OP2^+^ also showed connectivity to areas previously shown to activate following vestibular stimulation in a meta-analysis (Fig. 2) (zu Eulenburg et al. 2012). Subregions L1 and L3 connected with all meta-analysis regions (Fig. 3B, Fig. 2) (zu Eulenburg et al. 2012). Overall, 6 of 10 subregions in left OP2^+^ were connected to more than half of regions reported in the meta-analysis (L1, L3, L4, L8, L9, L10) (zu Eulenburg et al. 2012). L6 was largely connected with the occipital cortex. Subregions L5 and L7 had no meaningful gray matter connectivity (Fig. 3B).

**Fig. 4:**
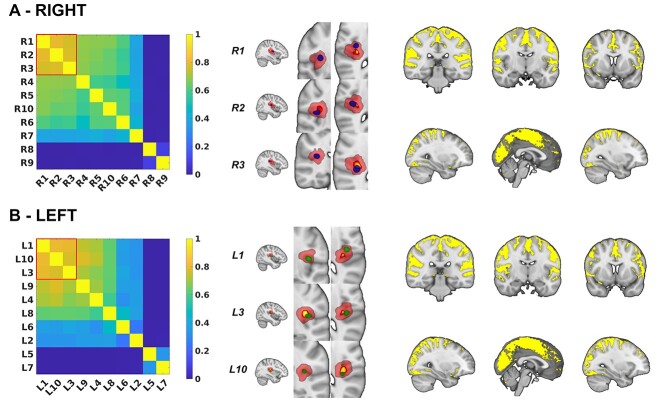
Common patterns of vestibular connectivity from OP2^+^ subregions. (A) Spatial similarity matrix for the whole-brain connectivity of right OP2^+^ subregions as measured by the Dice coefficient. Red square highlights 3 subregions with similar networks (R1 [anterior OP2^+^], R2 [lateral OP2^+^], and R3 [posterior OP2^+^]). These 3 subregions are illustrated in blue, on a background of the OP2^+^ mask in red. The core/central area of the OP2^+^ mask (*P* > 0.5 in Jülich atlas) is shown in yellow. Adjacent is a map of common functional connectivity from the 3 subregions (yellow, *P* < 0.05). (B) Spatial similarity matrix, OP2^+^ subregions (in green) and common functional connectivity for left OP2^ +^.

Hierarchical clustering was used to identify spatially similar OP2^+^ networks (Hastie et al. 2009). Three right OP2^+^ networks had high spatial similarity to each other (R1 [anterior OP2^+^], R2 [lateral OP2^+^], and R3 [posterior OP2^+^] (Dice coefficient > 0.7, Fig. 4A). Peak connectivity in this common network was with the primary somatosensory cortex, the parietal operculum (OP1 and OP2 bilaterally), supracalcarine cortex, left inferior parietal lobule, and the anterior cingulate. Analysis of left OP2^+^ connectivity showed less clear clustering of spatial similarity between whole-brain connectivity networks (Fig. 4B). However, the 3 most similar networks on the left shared functional connectivity to a network very similar to the common network identified from right OP2^+^ regions (Dice similarity co-efficient 0.80, Fig. 4).

### Bilateral OP2^+^ activation during vestibular stimulation

**Fig. 5:**
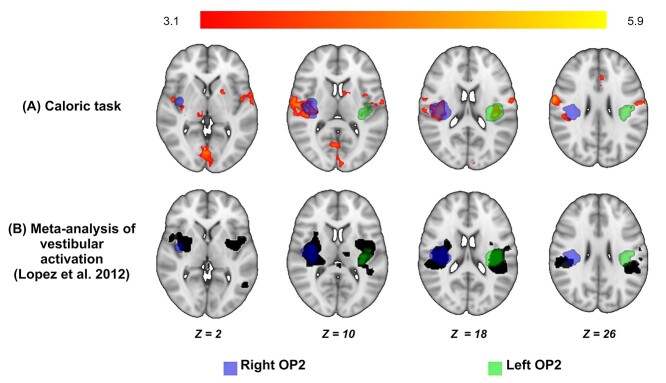
Bilateral OP2^+^ activation during caloric irrigation. (A) Areas of significant activation during caloric irrigation in healthy controls (red-yellow). Activation is seen in right and left OP2^+^. Masks of right (blue) and left OP2^+^ (green) are shown, defined using the Jülich atlas. (B). Vestibular activation produced in OP2^+^ by acoustic, caloric, or galvanic stimuli (black, courtesy of Dr Christophe Lopez, Aix-Marseille Université (Marseille, France); Lopez et al. 2012).

Caloric stimulation produced activation of OP2^+^ in both hemispheres in controls (Fig. 5). Right OP2^+^ showed more extensive activation than left OP2^+^ (right OP2^+^ peak: *x* = 38, *y* = −20, *z* = 17, 619 voxels; left OP2^+^ peak: *x* = −38, *y* = −26, *z* = 18, 219 voxels). Activation associated with caloric stimulation was also seen in the right precentral gyrus, intracalcarine cortex, left putamen, right thalamus, left thalamus, right frontal pole, and left lingual gyrus (Supplementary Tables S1 and S2). OP2 has previously been shown to be activated by caloric, galvanic, and acoustic stimuli. A recent meta-analysis (Lopez et al. 2012) has reported the average location of this activation, which we compared with activation produced during our caloric stimulation study. A similar pattern of OP2^+^ activation was observed with caloric stimulation and the meta-analysis of vestibular activation (Fig. 5).

### OP2^+^ responds to caloric irrigation but not after vestibular neuritis

**Fig. 6:**
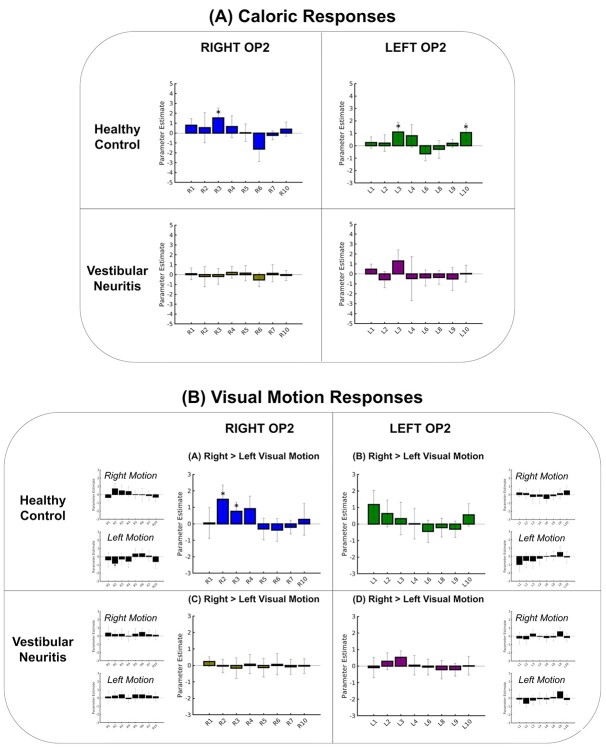
Caloric and visual motion responses in OP2^+^. (A) Caloric responses in healthy controls and patients with vestibular neuritis within right and left OP2^+^ subregions. (B) Visual motion responses in healthy controls and patients with vestibular neuritis. Larger bar charts show OP2^+^ subregion responses for motion selectivity (right > left visual motion). Smaller bar charts show specific responses to rightward and leftward motion (motion > static visual stimulation). Asterisk indicates significant activation relative to baseline, FDR-corrected.

Next, we investigated whether specific OP2^+^ subregions responded to caloric irrigation (of the left ear). In healthy controls, within the right hemisphere only right R3 [posterior OP2^+^] activated significantly during left ear caloric irrigation (*t*(16) = 3.33, *P* = 0.005, FDR-corrected *P* < 0.05, Fig. 6A). In the left hemisphere, L3 [lateral OP2^+^] and L10 [posterior OP2^+^] showed significant activation (*t*(16) = 3.18, *P* = 0.005; L10: *t*(16) = 3.23, *P* = 0.006, FDR-corrected *P* < 0.05, Fig. 6A). Patients with right vestibular neuritis showed abnormal vestibular responses in OP2^+^ (Fig. 6A). Caloric irrigation produced no significant activation of any OP2^+^ subregions in either hemisphere (Fig. 6A). R3 (posterior OP2^+^) showed significantly lower levels of activation in vestibular neuritis patients than controls (*t*(32) = 2.87, *P* = 0.007). In the left hemisphere, L10 [posterior OP2^+^] showed a reduction in activation in vestibular neuritis patients compared to controls of borderline significance (L10: *t*(32) = 1.98, *P* = 0.057). In summary, we identified 1 caloric-responsive subregion on the right (R3 [posterior OP2^+^]) and 2 on the left (L3 [lateral OP2^+^] and L10 [posterior OP2^+^]).

### Right OP2^+^ shows visual motion selectivity but not after vestibular neuritis

Visual motion activated both visual cortices (Supplementary Fig. S1 and Supplementary Table S3). Right OP2^+^ also responded selectively to the direction of visual motion in controls but not in patients with vestibular neuritis (Fig. 6B). In controls, R2 [lateral OP2^+^] activated during right visual motion and deactivated during left visual motion (right motion: *t*(16) = 2.41, *P* = 0.030, FDR-*P* > 0.05; left motion: *t*(16) = −3.28, *P* = 0.005, FDR-*P* < 0.05). Other subregions within right OP2^+^ did not show activation significantly modulated by motion. The direct comparison of responses to leftward and rightward visual motion showed that both right R2 [lateral OP2^+^] and R3 [posterior OP2^+^] showed motion selectivity in controls, responding significantly more to right than left visual motion (*t*(16) = 3.74, *P* = 0.002 and *t*(16) = 2.91, *P* = 0.011, respectively, Fig. 6B). No subregions responded more to leftward than rightward visual motion. No subregions in the left hemisphere showed directional responses to visual motion (Fig. 6B). This motion selectivity was not seen in patients with vestibular neuritis (Fig. 6B). No subregions in either hemisphere showed activity that was affected by motion. The direct comparison of controls and patients showed significantly lower motion selectivity in vestibular neuritis patients than in controls for both R2 [lateral OP2^+^] and R3 [posterior OP2^+^] (R2: *t*(32) = 3.37, *P* = 0.002; R3: *t*(32) = 2.32, *P* = 0.027, respectively).

### Activation of right OP2^+^ by caloric stimulation correlates with vertigo, nystagmus SPV, visual dependence, and disability

**Fig. 7:**
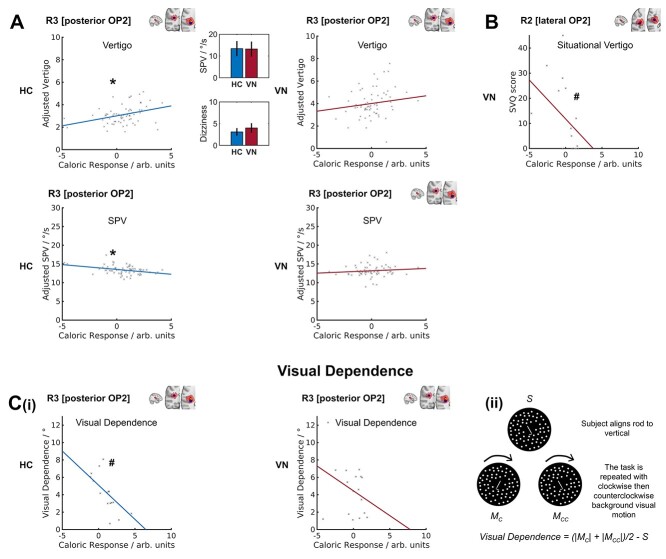
Correlation between subregion caloric response and vertigo, nystagmus SPV, visual dependence, and situational vertigo. (A) Scatter plots of vertigo and peak nystagmus SPV versus the caloric response in R3 [posterior OP2^+^] in healthy controls (HC) and patients with vestibular neuritis (VN); data points were obtained in each of 4 stimulus conditions. Inset bar charts show mean peak nystagmus SPV (in degrees per second) and mean vertigo; error bars show the 95% confidence intervals of the respective means. (B) Scatter plot of situational vertigo and R2 [lateral OP2^+^] caloric activation in VN; the line of best fit is for illustration only. (C) (i) Scatter plots of visual dependence and R3 [posterior OP2^+^] caloric response; (ii) illustration of rod and disc task from which visual dependence was calculated. The subjective visual vertical is measured when participants align a central rod overlying a static background (*S*); this is repeated during clockwise (*M_C_*) and counterclockwise (*M_CC_*) rotation of the visual background; visual dependence is calculated as shown. ^*^P < 0.05 for caloric response predictor; #*P* < 0.05 for Spearman correlation.

Caloric irrigation of the left ear produced subjective feelings of vertigo in controls and vestibular neuritis patients. More vertigo was reported in patients than controls (control mean 3.11, SD 1.95; patient mean 4.01, SD 2.53; *F*(1,131) = 5.52, *P* = 0.02, Fig. 7A). Adjusted vertigo was determined, which is a fitted value for vertigo predicted by the caloric response with other predictors averaged out. In controls, adjusted vertigo correlated with activation in R3 [posterior OP2^+^] (*t*(61) = 2.22, *P* = 0.030, Fig. 7A), but not other caloric-responsive OP2^+^ subregions in either the right or left hemispheres. OP2^+^ activation did not correlate with perceptions of vertigo in vestibular neuritis patients. Nystagmus during caloric stimulation can be quantified by its peak SPV (a measure of the vestibulo-ocular reflex). This did not differ between the groups (Fig. 7A) and did not correlate with vertigo in either group. Peak SPV did, however, correlate with less caloric activation in R3 [posterior OP2^+^] in controls (*t*(61) = −2.33, *P* = 0.023, Fig. 7A).

Individual differences in visual dependence also correlated with caloric responses in right posterior OP2^+^. Visual dependence was similar in controls and patients with vestibular neuritis (control mean 3.90°, SD 2.27°, patient mean 4.57°, SD 2.90°). However, a negative correlation between R3 [posterior OP2^+^] caloric activation and visual dependence was only significantly seen in controls (R3: Spearman *r* = −0.814, *P* < 0.001, Fig. 7C(i)). In patients with vestibular neuritis, OP2^+^ activation did not significantly correlate with visual dependence (Fig. 7C(i)).

The functional impact of dizziness and vertigo in patients with vestibular neuritis was measured using the DHI, SVQ, and VSS. There was no significant relationship between these measures and vestibular responses in caloric-responsive subregions: R3 [posterior OP2^+^], L3 [lateral OP2^+^], or L10 [posterior OP2^+^], which had activated in controls during caloric irrigation. We investigated whether a relationship existed in other subregions, which had similar whole-brain connectivity: R1 [anterior OP2^+^], R2 [lateral OP2^+^], and L1 [anterior OP2^+^] (Fig. 4). Higher activity in R2 [lateral OP2^+^] during caloric stimulation correlated significantly with lower SVQ scores (R2: Spearman *r* = −0.664, *z* = 2.99, *P* = 0.004, FDR-corrected *P* < 0.05, Fig. 7B). No other subregion responses correlated with SVQ scores. No significant correlations were found for DHI or VSS scores and subregion caloric activity. Left OP2^+^ caloric responses did not significantly correlate with any clinical symptom measures.

## Discussion

OP2 is a core region within the human cortical vestibular network on the basis of its location (Eickhoff, Schleicher, et al. 2006b; Eickhoff, Weiss, et al. 2006c), structural connectivity (Wirth et al. 2018; Indovina et al. 2020), and functional responses (Eickhoff, Weiss, et al. 2006c; zu Eulenburg et al. 2012). Here we define its functional anatomy by studying distinct patterns of connectivity measured using fMRI.

The human cortical vestibular network is involved in higher order vestibular functions such as the perception of self-motion, judgments regarding verticality, as well as balance control and spatial navigation (Lopez and Blanke 2011). OP2 is well connected to this network (zu Eulenburg et al. 2012; Wirth et al. 2018; Indovina et al. 2020), and the area’s centrality in the network predicts the existence of subregions further connected to vestibular brain areas involved in processing higher-order visuo-vestibular functions.

Our results indeed showed that functional connectivity in OP2^+^ echoes multiple whole-brain intrinsic connectivity networks, and the area has strong functional connectivity to a range of regions involved in vestibular function including the primary somatosensory cortex, the parietal operculum, the supracalcarine cortex, the left inferior parietal lobule, and the anterior cingulate cortex. We identify a subregion within posterior OP2^+^ (R3 [posterior OP2^+^]) responsive to vestibular and visual information and with activity correlated with self-motion perception, visual dependence, and peak nystagmus SPV. The pattern of functional connectivity we observed is similar to that seen in the nonhuman primate PIVC (Guldin and Grüsser 1998), a homologous area that also strongly connects to a number of cortical vestibular areas (Guldin et al. 1992; Guldin and Grüsser 1998). Our human results align with those from nonhuman primates and suggest that OP2 and its analogue the PIVC are heteromodal with respect to processing multisensory information relevant to higher order vestibular functions (Guldin and Grüsser 1998; Brandt and Dieterich 1999; Lopez and Blanke 2011).

The role of OP2^+^ in processing visual motion information, however, has been contentious (Grüsser et al. 1990a; Brandt et al. 1998; Dieterich et al. 1998; Chen et al. 2010; Billington and Smith 2015; Chen et al. 2016; Frank, Wirth, et al. 2016b). Key evidence against the processing of visual information stems from a study that showed no neural response in the macaque PIVC to moving dot stimuli (Chen et al. 2010). Chemical deactivation of anterior or posterior subregions of the macaque PIVC, however, impairs perceptual judgments of head orientation informed by visual cues (Chen et al. 2016), suggesting the PIVC processes visual motion information. In humans, parieto-insular areas including OP2 deactivate in response to visual motion (Kleinschmidt et al. 2002; Laurienti et al. 2002), particularly when it is the focus of attention (Frank, Sun, et al. 2016a; Frank et al. 2020, 2021). Activation is instead seen in the retroinsular cortex (Brandt et al. 1998; Sunaert et al. 1999; Orban et al. 2003; Frank et al. 2014; Frank, Wirth, et al. 2016b). Here we showed a posterior ICA-derived subregion of right OP2^+^ (R3 [posterior OP2^+^]) extending into the anterior retroinsular cortex processes visual information, given its directional responses to visual motion stimuli and correlation between vestibular activity and visual dependence. Our results support the proposition that visuo-vestibular processing mainly occurs in the retroinsular region at the posterior border of OP2 (Frank and Greenlee 2018), and also suggest this processing may extend into OP2. Additionally, our finding of correlation between more activity in this subregion and lower peak nystagmus SPV suggests OP2 may be implicated in the processes, which mediate top-down control of brainstem vestibulo-ocular reflex responses (Arshad et al. 2013).

There has also been debate about whether vestibular processing is lateralized in OP2 (Dieterich et al. 2003; Brandt and Dieterich 2015; Kirsch et al. 2018; Wirth et al. 2018; Indovina et al. 2020; Raiser et al. 2020). The cytoarchitecture of OP2 is symmetrical (Eickhoff, Schleicher, et al. 2006b). Studies of the region’s structural connectivity have shown mixed results with right (Wirth et al. 2018; Raiser et al. 2020) or left-lateralized patterns of cortico-cortical connectivity (Indovina et al. 2020). A functional connectivity study showed OP2 and neighboring regions had both symmetric and lateralized cortico-cortical connectivity (Kirsch et al. 2018). Studies of cortical responses to vestibular stimuli have shown the right hemisphere (and thus right OP2) is dominant for vestibular functions (Dieterich et al. 2003; Dieterich and Brandt 2008). We found no evidence of lateralization in the pattern of functional connectivity in OP2^+^ subregions (Fig. 4) or the response to caloric stimuli (Fig. 5). However, the effects of peripheral vestibular disease were asymmetrical and the relationship between activity and vertigo/visual dependence were only seen in right OP2^+^ (Fig. 7A and C(i)), suggesting higher-order vestibular functions lateralize to the right hemisphere, as in handedness-related vestibular lateralization (Arshad et al. 2013, 2019; Chan et al. 2021).

Interestingly, OP2^+^ responses to vestibular and visual stimuli were influenced by vestibular disease. In patients with vestibular neuritis, right OP2^+^ responses to caloric stimulation (of the healthy ear) and visual stimuli were abolished, supporting a role for right OP2^+^ in the normal processing of vestibular and visual stimuli. A recent study of cortical responses to galvanic stimuli in patients with bilateral vestibular failure found no relationship between dizziness handicap and activity in OP2 (Helmchen et al. 2019), and in our results the relationship between OP2^+^ activity and visual dependence seen in healthy subjects was lost in vestibular neuritis patients. In addition, we observed that greater visually induced dizziness (SVQ) in patients correlated with less vestibular activity in a distinct lateral subregion of right OP2^+^ (R2 [lateral OP2^+^]). This suggests that reduced vestibular responses in OP2^+^ may be a marker of a poorer clinical outcome.

Our finding of a relationship between OP2^+^ activation and chronic visually induced dizziness following vestibular neuritis is in keeping with findings across recent neuroimaging studies that have revealed altered OP2^+^ function and connectivity across a range of central vestibular disorders. For example, changes in task-free functional connectivity have been reported between OP2 and a range of other regions in persistent postural-perceptual dizziness (PPPD) (Lee et al. 2018; Popkirov et al. 2018; Li et al. 2020) with reduced connectivity to multisensory areas consistently reported, which may reflect maladaptive integration of vestibular information (Im et al. 2021). In vestibular migraine, the left PIVC has been reported to show less gray matter volume and more functional connectivity with other somatosensory areas (Zhe et al. 2021) and, in patients with post-concussive visual motion sensitivity, increased activation in OP2 is observed following “provocative” visual stimuli (Allen et al. 2021). Taken together, these findings suggest altered OP2^+^ connectivity is common across a range of central vestibular disorders, and these abnormalities may cause chronic vestibular symptoms.

The change in OP2^+^ activity in vestibular neuritis we identified occurred despite similar vestibulo-ocular reflex function across patients (following stimulation of the healthy ear) and controls. These results align with previous work showing cortical vestibular processing can be modified separately from brainstem reflex function (Okada et al. 1999; Nigmatullina et al. 2015). Changes in cortical vestibular processing have been suggested to be relevant to adaptation and recovery following vestibular disease (Palla et al. 2008; Seemungal 2014; Yip and Strupp 2018). However, the mechanism underpinning reduced vestibular responses in OP2^+^ to stimulation of the healthy ear is unclear. One possible mechanism is that there may be reduced vestibular input to OP2^+^ following vestibular neuritis. This implies reduced vestibular signaling, separable from preserved vestibulo-ocular reflex functioning. Such reduced vestibular signaling may underpin the increase in visual dependence in patients. Animal studies have shown unilateral vestibular loss leads to distinct effects within the brainstem and that the correlates of nystagmus and behavioral recovery differ (Smith and Curthoys 1989). In contralesional medial vestibular nuclei, rapid restitution of resting neural activity occurs over a similar time frame to nystagmus recovery, whereas desensitization to vestibular signals recovers over a slower time frame in parallel with behavioral recovery (Markham et al. 1977; Yagi and Markham 1984; Smith and Curthoys 1988). To our knowledge, the cortical correlates of these changes have not previously been studied. Our findings in OP2^+^ may be a correlate of such desensitization.

Our study has a number of limitations. First, our comparisons operate on the assumption that OP2^ +^’s subregional anatomy is invariant between controls and patients. Reports of structural and functional changes in the vicinity of OP2 following peripheral vestibular disease (Helmchen et al. 2009, 2014) and in association with central vestibular disorders such as PPPD (Im et al. 2021) and vestibular migraine (Zhe et al. 2021) mean this assumption may not have been satisfied. Group differences, if present, may have influenced correlations between neural activity in OP2^+^ and vestibular functions such as self-motion perception and visual dependence. Second, OP2^+^ activations in patients following caloric stimulation were weaker than in controls; this may have contributed to nonsignificant correlation between activation and higher vestibular function due to floor effects. Third, spatial smoothing in spatially constrained ICA may have increased the apparent spatial extent of functional subregions. Fourth, the results of our analyses depend on dimensionality in spatially constrained ICA. We maximized between-subject reproducibility to obtain an optimal number of dimensions for ICA in a data-driven way (Moher Alsady et al. 2016). In right OP2^+^, the mean reproducibility curve had a global maximum at ten subregions. The data thus provided a clear justification for our choice. Notably, though a few subregions had similar whole-brain connectivity (Fig. 4), they nonetheless had distinct task-related activation, confirming good separability (Fig. 6).

In summary, our results show a posterior subregion in right OP2^+^ (R3 [posterior OP2^+^]) is well connected to other areas in the cortical vestibular network, and the subregion processes visual and vestibular information relevant to the perception of self-motion and verticality; the subregion may also be involved in the top-down control of the vestibulo-ocular reflex. We also find that vestibular responses in a different subregion (R2 [lateral OP2^+^]) are variably affected by chronic vestibular neuritis, and this effect appears relevant to symptomatic recovery.

## Supplementary Material

Supplementary_bhac085
