# Supplementary material for: The human vestibular cortex: functional anatomy of OP2, its connectivity and the effect of vestibular disease
Source: Cereb Cortex. 2022 Mar 2;33(3):567–82. doi: 10.1093/cercor/bhac085 (PMC9890474; doi:10.1093/cercor/bhac085)
Supplement: Supplementary_bhac085 [file supplementary_bhac085.docx]

Supplementary material

# Results

## Bilateral OP2 activation during vestibular stimulation

### Group-level caloric activation results for controls

The table below is an output of FEAT (within FSL (Jenkinson et al. 2012)) showing significant clusters of activation at the group level. Z-MAX is the maximum z-score. COPE = Contrast of parameter estimates.

| Cluster Index | Voxels | P | Z-MAX | Z-MAX X (mm) | Z-MAX Y (mm) | Z-MAX Z (mm) | Z-COG X (mm) | Z-COG Y (mm) | Z-COG Z (mm) | COPE-MAX | COPE-MAX X (mm) | COPE-MAX Y (mm) | COPE-MAX Z (mm) | COPE-MEAN |
| --- | --- | --- | --- | --- | --- | --- | --- | --- | --- | --- | --- | --- | --- | --- |
| 8 | 12742 | 0 | 5.69 | 40 | -18 | 48 | 8.31 | -18 | 48.2 | 53.3 | 46 | -14 | 60 | 19.9 |
| 7 | 1126 | 8.13E-10 | 4.64 | 2 | -78 | 4 | -5.14 | -85.6 | -0.542 | 38.2 | -22 | -90 | -18 | 21.1 |
| 6 | 507 | 1.54E-05 | 4.49 | -18 | 10 | -6 | -20 | 6.14 | -6.12 | 40.3 | -8 | 4 | 8 | 21.7 |
| 5 | 452 | 4.35E-05 | 4.56 | 20 | -28 | -4 | 18.9 | -26.3 | -6.88 | 33.6 | 12 | -32 | -4 | 18.1 |
| 4 | 223 | 0.00571 | 4.99 | -38 | -26 | 18 | -38.6 | -20.6 | 17.6 | 28.5 | -38 | -28 | 18 | 16.8 |
| 3 | 211 | 0.00765 | 4.41 | -14 | -30 | 0 | -18.6 | -31 | -3.05 | 27.6 | -12 | -34 | -2 | 16.9 |
| 2 | 191 | 0.0126 | 3.77 | 20 | 46 | 26 | 22.5 | 45.2 | 31.3 | 15.8 | 22 | 44 | 34 | 12.4 |
| 1 | 146 | 0.0413 | 3.88 | -22 | -58 | 2 | -17.5 | -64.6 | 9.01 | 22.1 | -16 | -62 | 8 | 15.8 |

**Table 1 – Significant clusters of activation during caloric stimulation in controls**

The regions of highest probability represented by the centre of gravity results (Z-COG) were identified within the Harvard-Oxford Cortical Structural Atlas and are summarised for each cluster in the table below.

| Cluster index | Location | Atlas |
| --- | --- | --- |
| 8 | Right Precentral gyrus | Harvard-Oxford |
| 7 | Intracalcarine cortex | Harvard-Oxford |
| 6 | Left putamen | Harvard-Oxford |
| 5 | Right thalamus | Harvard-Oxford |
| 4 | Left parietal operculum | Harvard-Oxford |
| 3 | Left thalamus | Harvard-Oxford |
| 2 | Right frontal pole | Harvard-Oxford |
| 1 | Lingual gyrus | Harvard-Oxford |

**Table 2 – Matching brain areas for each cluster of activation during caloric irrigation in controls**

## Whole brain response to visual motion

**
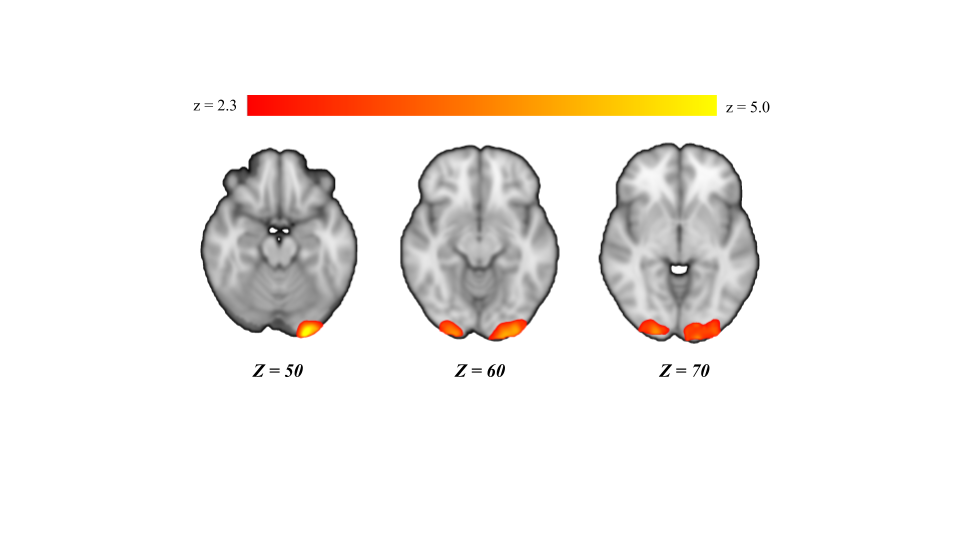
**

**Figure 1 - Visual cortices activate during visual motion.**

Results are based on FEAT (within FSL (Jenkinson et al. 2012)) for a contrast of visual motion (L + R - Static). The cluster forming threshold was z = 3.1 with family wise error rate p < .05.

| Cluster Index | Voxels | P | -log10(P) | Z-MAX | Z-MAX X (mm) | Z-MAX Y (mm) | Z-MAX Z (mm) | Z-COG X (mm) | Z-COG Y (mm) | Z-COG Z (mm) | COPE-MAX | COPE-MAX X (mm) | COPE-MAX Y (mm) | COPE-MAX Z (mm) | COPE-MEAN |
| --- | --- | --- | --- | --- | --- | --- | --- | --- | --- | --- | --- | --- | --- | --- | --- |
| 2 | 1256 | 3.34e-06 | 5.48 | 5.45 | -24 | -100 | -18 | -23.1 | -96.6 | -9.72 | 96.9 | -26 | -96 | -12 | 55.8 |
| 1 | 670 | 0.000523 | 3.28 | 4.4 | 22 | -100 | -8 | 25.7 | -95.1 | -6.03 | 105 | 30 | -92 | -10 | 65.8 |

**Table 3 – Significant clusters of activation during visual motion in controls**

##

# References

Jenkinson M, Beckmann CF, Behrens TEJ, Woolrich MW, Smith SM. 2012. FSL. Neuroimage. 62:782–790.
